# Stress and retention challenges among rural and regional physicians: a mixed-methods systematic review and framework for action

**DOI:** 10.1093/pubmed/fdag011

**Published:** 2026-02-15

**Authors:** Joëlle V F Coumans, Ilona Ciller

**Affiliations:** University of New England, School of Rural Medicine, Elm Avenue, Armidale, NSW 2351, Australia; Affiliated with the University of Newcastle College of Health, Medicine and Well-being, University Drive, Callaghan, NSW 2308, Australia; University of New England, School of Rural Medicine, Elm Avenue, Armidale, NSW 2351, Australia; Affiliated with the University of Newcastle College of Health, Medicine and Well-being, University Drive, Callaghan, NSW 2308, Australia

**Keywords:** rural physicians, burnout, occupational stress, mental health, workforce retention

## Abstract

**Introduction:**

Workplace stress among rural physicians is a pressing public health challenge, intensified by increasing workloads, demographic shifts, and constrained healthcare infrastructures. This systematic review examines the stress-related outcomes rural physicians face, identifies key contributing and mitigating factors, and proposes a transformative framework for sustainable intervention.

**Methods:**

A comprehensive search across five databases (January 2020–2025) yielded 1973 studies, with 24 meeting inclusion criteria focused on rural, remote, or regional physicians. Data were synthesised using PRISMA guidelines and quality-assessed with standardised checklists.

**Results:**

Across 11,130 rural physicians, burnout emerged as the most prevalent outcome. Excessive workloads, diminished autonomy, blurred work-life boundaries, and systemic under-resourcing drove emotional exhaustion (EE). Geographic isolation further compounded anxiety, depression, and sleep disruption. While job dissatisfaction led to absenteeism and turnover, protective factors included professional autonomy, recognition, and task diversity. Promising interventions included work-life balance strategies, continuous education, and context-responsive recruitment policies.

**Conclusions:**

Sustaining rural healthcare requires more than short-term solutions; it calls for systemic reform that centres physician well-being, autonomy, and community-rooted support. Equity-driven frameworks anchored in self-actualisation, collaboration, and culturally responsive remote care offer promising paths forward. Future research must prioritise context-specific, structural change across diverse rural landscapes.

## Introduction

Workplace stress, a systemic issue, disproportionately affects physicians, resulting in higher rates of psychological distress and burnout. This work-related syndrome is defined by high emotional exhaustion (EE), feelings of cynicism/depersonalisation, and a sense of professional inefficacy.^1^ Over time, physician stress has shifted from the relational weight of the doctor-patient interaction to structural pressure demands. These are best understood through established occupational stress models, such as the Job Demands-Resources (JD-R) model, which frames stress as an imbalance between high job demands (e.g. proliferating bureaucracy, excessive workload) and low resources (e.g. professional autonomy, poor support).^2^^,^^3^

These shifts are more acutely felt in rural and remote regions, where physician shortages are twice the urban rate.^4^ For consistency, this manuscript uses 'rural' as the primary descriptor for all non-metropolitan settings. This disparity reflects chronic underinvestment, uneven infrastructure, and longstanding neglect of rural health systems.

A demographic shift (e.g. a decline from 52% to 43% of the global rural population), is often used to justify health services centralisation.^5^ Coupled with widening disparities in health social determinants and an aging population with a higher chronic disease burden, this has exacerbated rural/urban inequities.^6^^,^^7^ Addressing these issues is complex, evident in large-scale health system reforms, such as those ongoing in China.^8^ Disproportionately, these inequities affect Indigenous and marginalised communities, adding to the strain on the few physicians serving under-resourced rural areas.^9–12^

In rural practice, physicians fulfil multiple roles with overlapping responsibilities beyond usual clinical duties, heightening emotional strain. Isolation, both geographic and professional, further amplifies these pressures as rural physicians typically have less access to specialist support, administrative assistance, or collegial networks.^6^ These gaps are closely associated with psychological distress, including burnout,^13^ depression,^14^ and in some cases, suicidal ideation.^15^^,^^16^

Beyond geography, rural practice challenges include structural and professional constraints that discourage open discussion of distress. Long working hours, limited financial incentives, and frequent on-call duties are well-documented contributors to job dissatisfaction and emotional fatigue.^17^ This dynamic reflects an *'Effort-Reward Imbalance*,' where high effort is met with inadequate rewards (e.g. salary, esteem, or security), causing distress. This is amplified by '*overcommitment,*' common in medicine,^18^^,^^19^ and in rural areas due to understaffing, policy neglect, and a workplace culture that discourages vulnerability.

The COVID-19 pandemic further amplified these pressures by exposing underlying vulnerabilities in healthcare work beyond biomedical risks. Even if rural regions experienced lower case numbers,^20^ physicians experienced substantial psychological strain. Reports of presenteeism, ‘*quiet-quitting,*’ absenteeism, and high turnover intentions surged,^21–24^ affecting continuity of care, eroding patient trust, and the community-specific knowledge physicians provide.

In many rural communities, physicians often provide care within overlapping social networks, which can lead to blurred professional boundaries, such as treating acquaintances, neighbours, or extended family members.^25^ These dynamics may contribute to additional emotional demands and ethical complexity, particularly without formal guidance or institutional support.

Given the rural–urban disparities, focusing only on increasing physician numbers is insufficient.^26^ Workforce initiatives, such as recruitment programs or financial incentives, often emphasise supply but neglect the working conditions necessary for retention and well-being. In response, it has been proposed to strengthen rural practice environments through culturally competent team-based care, reforms in medical education, and addressing physician migration.^27^

Accordingly, this review is guided by Carol Weiss’ Theory of Change framework, which emphasises understanding of how and why outcomes occur through defined causal pathways.^28^^,^^29^ This framework is particularly suitable for rural healthcare, where multiple interacting variables can shape individual experiences.

Although stress and mental health challenges among healthcare professionals are widely studied, no previous reviews have examined how personal, professional, organisational, and community stressors collectively impact physician well-being, diminishing professional efficacy, and prompting physicians to leave.

**Figure 1:**
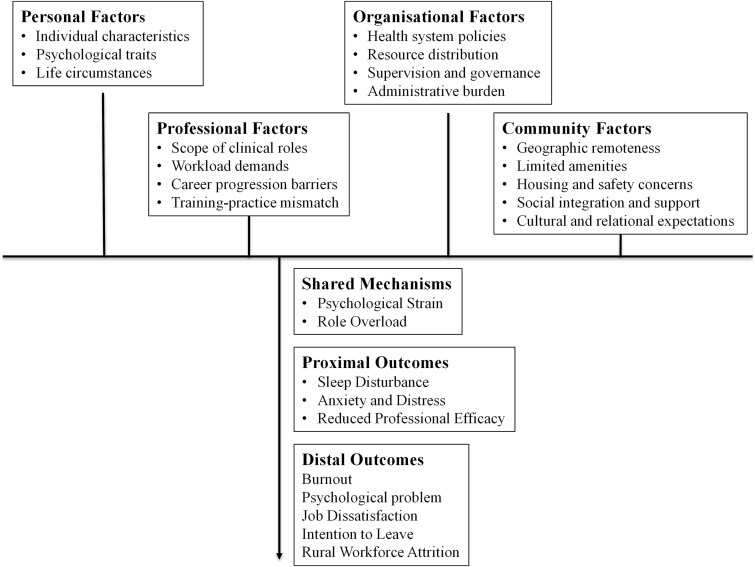
Conceptual framework illustrating pathways from multi-level stressors to workforce outcomes, informed by Carol Weiss’ theory of change.

Guided by this, we structured this mixed-studies systematic review to map proximal and distal outcomes (Fig. 1). Applying a narrative synthesis, we grounded the frameworks of the JD-R model,^2^ the Effort-Reward Imbalance model,^18^ and Conservation of Resources theory^30^ to integrate recent evidence on stress-related outcomes among rural physicians to address this knowledge gap.

The specific aims are to:

1. Identify the most prevalent stress-related outcomes among rural physicians.
2. Map key personal, professional, organisational, and community-level contributors to these outcomes.

Highlight evidence-informed strategies that support physician retention, well-being, and sustainable healthcare delivery in underserved settings.

No published or registered (PROSPERO) (updated May 27, 2024) reviews have examined this set of factors.

Addressing this gap is essential to inform policies and interventions that reflect the lived realities of rural healthcare delivery, as an integrated understanding can contribute to effective, context-sensitive strategies that promote physician well-being and system sustainability.

## Methods

### Eligibility criteria

This review followed a Population–Exposure–Outcome (PEO) framework:

- **Population**: Physicians (clinicians and doctors) practising in remote, rural, or regional areas. Studies involving mixed healthcare populations were included only if data specific to rural physicians were reported.
- **Exposure**: Work-related and personal stressors affecting mental health and well-being.
- **Outcomes**: Stress-related outcomes including burnout, EE, job dissatisfaction, anxiety, depression, sleep disturbances, presenteeism, absenteeism, and intention to leave.

Eligible studies were original, peer-reviewed research (qualitative, quantitative, or mixed-methods), published in English between January 2020 and January 2025. We excluded reviews, conference abstracts and opinion pieces (Supplemental Data S1, reason 1), studies focused exclusively on urban settings (Supplemental Data S1, reason 2), interventions lacking rural-specific physician data (Supplemental Data S1, reason 3), and, to avoid transient pandemic-specific bias that confounds factors for long-term retention in stable practice, those on COVID-19 (Supplemental Data S1, reason 4).

### Search strategy and study selection

Five electronic databases were searched: Embase, PsycINFO, Scopus, Web of Science, and PubMed initially in April 2024, and updated on January 6, 2025. Search strategies combined keywords related to rural healthcare, physician stress, and mental health outcomes (Supplemental Data S2).

Search results were imported into Covidence to perform an independent dual screening of titles, abstracts, and full texts. Disagreements were resolved through discussion and consensus. Reference lists of included studies were hand-searched to identify additional relevant articles.

### Data extraction and quality appraisal

A standardised form was developed to extract data on study characteristics, methods, findings and quality assessment (Supplemental Data S3).

Studies were appraised using the validated appraisal tool QuADS,^31^ which is designed to include heterogeneous studies (Supplemental Data S4).

### Data synthesis

Given the diversity of study designs, we used a thematic analysis and an integrative narrative synthesis. Extracted Data (Supplemental Data S3) were first tabulated under predefined domains guided by Carol Weiss’ Theory of Change.^28^^,^^29^ This included personal, professional, organisational, and community-level factors, alongside proximal (e.g. anxiety, sleep disturbance) and distal outcomes (e.g. burnout, job dissatisfaction).

Two additional models: self-actualisation principles^32^^,^^33^ and the Five Domains of Well-Being^34^ informed interpretation and the development of a conceptual framework for potential intervention pathways.

## Results

### Study selection and characteristics

From 1,973 records, 1,109 titles and abstracts were screened. Of these, 1,076 were excluded based on predefined criteria. Thirty-three full-text articles were reviewed, and 22 studies met eligibility requirements (Supplemental Data S1, S2, S5).

This included research conducted in 10 countries, primarily China (n = 7) and Canada (n = 4). likely reflecting the high volume of scholarship surrounding China's major health system reforms.^8^

In total, this study captured the perceptions of 11,130 rural physicians working in hospitals,^35–38^ general practices,^39–49^ or in village and township clinics^50–54^ or remote islands.^55^

### Stress-related outcomes

Stress-related outcomes among rural physicians were grouped into three main categories:

*Burnout,* ^35^ ^,^ ^36^ ^,^ ^39^ ^,^ ^42^ ^,^ ^43^ ^,^ ^45^ ^,^ ^46^ ^,^ ^48^ ^,^ ^49^ ^,^ ^52–54^ particularly EE^35^^,^^36^^,^^46^ was a frequently reported outcome across settings. Although prevalence rates in these studies varied, they are comparable to those of urban physicians,^39^^,^^43^^,^^48^ suggesting this is a widespread issue shaped by contexts.

*Mental health outcomes*, including anxiety, depression, and sleep disturbance, were frequently reported.^35^^,^^36^^,^^46^^,^^50^^,^^55^ Some studies noted higher anxiety rates among physicians working in isolated settings or those in sole clinical roles.^36^^,^^55^ Several studies reported depressive symptoms, with estimates ranging from 35.6% to 49%.^36^^,^^46^^,^^50^ Work-related sleep disturbance associated with a higher likelihood of EE was also noted, particularly among physicians in training.^35^

*Professional disengagement* varied across contexts and was demonstrated by a decline in job satisfaction, presenteeism, absenteeism, and intentions to reduce clinical hours or to leave.^37^^,^^38^^,^^44^^,^^50^^,^^51^^,^^56^

These findings suggest that stress-related outcomes reflect systemic issues rather than individual factors alone.

### Intersecting factors: a multi-level view

The key findings are summarised in Table 1 and detailed in Supplemental Data S3.

**Table 1:** Summary of contributing and mitigating factors across the domain.

Domain	Category	Contributing factors	Mitigating factors
Personal	Individual traits	Neuroticism, alexithymia, difficulty displaying weakness	Resilience, intellectual curiosity, and skills development
	Health & demographics	Sleep disturbance, presenteeism, health problems, gender, age, and nationality	Part-time work, flexible scheduling
Family	Work-life conflict	Partner dissatisfaction, childcare, family illness, desire to be near extended family	Supportive family integration, partner engagement
Professional	Role expectations	Solo practice, heavy patient loads, overtime, inability to take leave, complexity	Autonomy, focus on clinical tasks, and professional control
	Career development	Lack of continuing professional development, perceived inequities in promotion and pay, and inadequate mentoring	Career opportunities, recognition, and advanced rank, effort-reward balance
Organisational	Systemic barriers	Administrative burden, poor staffing, reliance on locums, racism, unfair workload	Supportive leadership, debriefing, fair pay, control over rest/schedule
Community	Social integration	Isolation, cultural/language barriers, personal privacy loss, gossip, community violence	Strong social/community ties, valued relationships
	Infrastructure	Lack of amenities, housing, and partner job access	Access to amenities & safe housing, nature/outdoor lifestyle

#### Contributing factors

Factors that drive stress-related outcomes for hospital physicians arise from organisational, systemic, and individual issues. At the organisational level, drivers include excessive workloads and administrative burden, a lack of management support, inadequate remuneration, and sub-standard equipment.^36^^,^^38^ Stress is also compounded by system-level challenges, ranging from unsafe working environments to community violence.^37^^,^^38^ A lack of career progression,^36^ was reported, with some systems supporting physicians with socio-political connections.^38^ Finally, individual factors, e.g. being female, a junior doctor, or facing personal health challenges, are also associated with higher stress.^35^^,^^36^

Comparative studies report similar burnout prevalence between urban and rural settings,^39^^,^^43^^,^^48^ suggesting context- or stressor-specific challenges for retention. This complexity is highlighted by one study showing that rural GPs can experience lower job stress but a higher intent to leave.^40^ Key challenges leading to burnout and lifestyle-driven turnover include unsustainable workloads and social factors, such as family commitments and a lack of partner career prospects.^44^^,^^45^ For internationally educated physicians, this is compounded by professional frustrations, like being directed into lower-prestige roles, which fosters a sense of powerlessness.^41^ Furthermore, some settings are characterised by community violence and a lack of cultural safety, most prevalent within remote Indigenous contexts.^42^^,^^45^

For the seasoned but under-resourced rural physicians of East Asia, stress-related outcomes are associated with structural pressures leading to psychological strain (47, 50, 51, Zhao, 2021 #23, 52, 54–56). These pressures include excessive workloads, a low-reward environment defined by low income, weak doctor-patient relationships and limited institutional support.^50^^,^^51^^,^^53^^,^^54^^,^^56^ This combination of factors manifests as depressive symptoms,^50^ EE from *'surface acting,*'^47^ and the anxiety of professional isolation.^55^ Ultimately, alongside factors like low job satisfaction, poor resilience, and personal traits like alexithymia, lead to burnout and turnover intention.^51^^,^^52^

#### Mitigating factors

For physicians working in hospitals, several key factors mitigate stress-related outcomes, with organisational and social support being most protective. Supportive management alongside manageable workloads and a work-life balance reduces stress.^35^^,^^36^ Socially, a positive atmosphere and strong collegial relationships increase personal accomplishment (PA) and mitigate isolation, while good local community relationships help reduce turnover.^36^^,^^38^ Finally, at the individual level, opportunities for career development lower stress.^36^

For rural physicians, job satisfaction is enhanced when supportive management provides autonomy to practice in alignment with professional values and supports continuing education.^49^ For South African physicians in Canada, retention occurs when the rural role enables them to achieve upward professional mobility and fulfil migration aspirations through focused clinical and administrative opportunities.^41^ Strong social and peer support is an answer to clinical isolation.^45^^,^^49^ Individual factors like part-time work,^43^ and the ability to have autonomy and control over one's work, including the flexibility to scale back hours, protect against burnout.^39^ However, these professional supports are often insufficient to overcome social and familial pressures. Retention is highly dependent on familial factors, most notably the lack of career prospects for a physician's partner and a failure to integrate socially into the community, leading to feelings of being a perpetual 'outsider' and living 'under a microscope.'^44^^,^^45^ This reveals a crucial distinction: while a positive practice environment is key to mitigating stress, it is often the unique social and familial challenges of the rural setting itself that are decisive for retention.

For the physicians of rural East Asia, protective factors shift towards issues of reward and professional dignity. A balanced effort-reward dynamic, with fair compensation for the work performed,^50^^,^^53^ and clear opportunities for professional advancement,^56^ are key protectors against burnout and depressive symptoms and retention, respectively. This could be reinforced by comprehensive support systems, including direct institutional backing from higher-level facilities^54^ interprofessional collaboration to ease professional isolation,^55^ and positive doctor-patient relationships at the community level.^53^ Finally, psychological strength arises from the ability to focus on core medical tasks over administrative duties.^54^ This is supported by findings that high job satisfaction and resilience foster strong work engagement, protecting against turnover,^51^ while a strong sense of occupational commitment buffers against the EE leading to turnover intention.^47^ Therefore, the core challenge in this context is to ensure that the role is structurally viable and professionally rewarding enough to be sustainable.

### Contextual variation

While common themes emerged, contextual variation, e.g. health system type, career stage, gender, and practice structure, also emerged. In Bangladesh and South Africa, early-career physicians working in under-resourced health systems and mandatory rural service programs reported extreme workload, infrastructure deficits, and safety concerns.^36–38^^,^^46^ In China, mid-career rural physicians, often village doctors with limited formal training, report working in isolation and carrying administrative burdens, contributing to EE and turnover intention.^47^^,^^51^^,^^52^^,^^54^^,^^56^

In contrast, in high-income systems such as Canada, Australia, the United States, and Scotland, stress was more often linked to high clinical demands, limited career progression, and professional isolation.^39^^,^^41^^,^^43–45^^,^^48^^,^^49^ Meanwhile, in centralised or well-resourced systems such as Japan, Saudi Arabia, and Sweden, stress reflected role expectations, limited autonomy, and hierarchical structures rather than material scarcity.^55^

Across settings, early-career physicians reported anxiety, reduced confidence, and stronger intentions to leave, especially when working without adequate supervision or peer support, particularly those in solo or mandatory placements.^35^^,^^36^^,^^38^^,^^46^^,^^55^ Mid- and late-career physicians reported emotional fatigue linked to sustained role complexity, reduced autonomy, and the erosion of professional identity in the face of growing administrative and clinical demands.^45^^,^^49^^,^^52^^,^^56^ Gender differences were also noted; e.g. in South Africa, female physicians reported higher levels of burnout due to workload and safety concerns.^36^^,^^46^ Regardless of a country's income level, stress occurs more in isolated or under-supported practice settings.

## Discussion

### Main finding of this study

This systematic review identified interrelated stress-related outcomes: burnout^35^^,^^36^^,^^39^^,^^42^^,^^43^^,^^45^^,^^46^^,^^48^^,^^49^^,^^52–54^ adverse mental health,^35^^,^^36^^,^^46^^,^^50^^,^^55^ professional disengagement, which is evidenced by absenteeism and high turnover intention.^37^^,^^40^^,^^44^^,^^47^^,^^51^ These issues manifest as high EE, depression, and a diminished sense of PA.^36^^,^^46^^,^^52^ This results in an erosion of professional identity, as evidenced by physicians’ frustrated career aspirations and a loss of professional values.^41^^,^^49^ A combination of high workloads,^49^^,^^50^ inadequate remuneration,^53^^,^^56^ limited career progression,^41^ and personal factors, such as the lack of partner career opportunities, lead to turnover intention^47^ and strained patient relationships.^53^

### What is already known on this topic

Burnout is a well-documented challenge across the healthcare workforce globally^57–62^ associated with reduced engagement^63^ increased safety incidents, declining trust^64^^,^^65^ and a substantial economic burden.^66^^,^^67^

Burnout, when compounded with high workloads, erratic schedules, and delayed leave, affects well-being.^68^^,^^69^ Supportive work environments, marked by autonomy, empathy, equity, and opportunities for growth, can mitigate these effects.^70^^,^^71^ Inclusive leadership, flexible scheduling, and collegial teamwork are especially protective.^72^

In rural settings, additional community-based strategies, such as social belonging, access to childcare, education, and partner employment, were also cited for retention.^73–75^

### What this study adds

This review synthesises cross-cultural evidence demonstrating how a lack of progress towards career goals,^41^^,^^56^ and a loss of professional autonomy^42^^,^^45^^,^^49^ are key factors of burnout and turnover intention.

**Figure 2:**
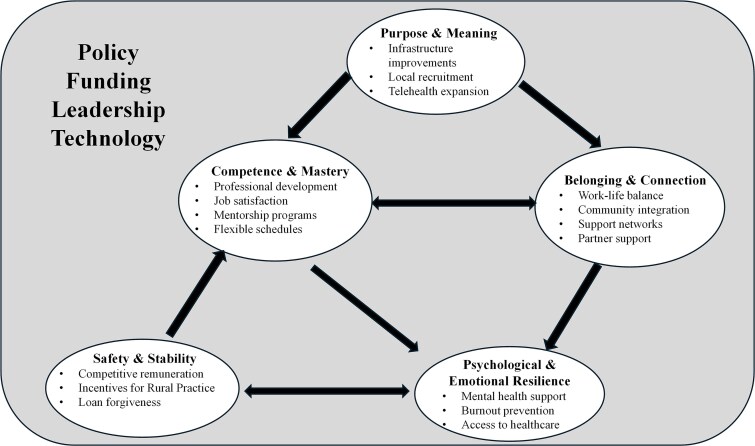
Integrated framework around the five essential elements of well-being and their possible impact on rural physician retention.

To address this multifaceted challenge, we propose a disruptive retention framework that builds on established models for rural retention, emphasising organisational strategy,^76^ a sense of belonging,^73^ and physician identity,^74^ with internal physicians' psychological needs, e.g. well-being domains,^34^ and self-actualisation principles^32^^,^^33^ (Fig. 2). Our framework directly operationalises our findings (Supplemental Data S3) by prioritising five interconnected domains designed to challenge the status quo and foster physician commitment: Safety & Stability through equitable remuneration/fair working conditions^46^^,^^50^^,^^56^; Belonging & Connection via strong community/peer support,^37^^,^^42^ Competence & Mastery via career development/mentorship,^41^^,^^49^ Purpose & Meaning by empowering physicians as community leaders^42^^,^^54^^,^^56^ and Psychological & Emotional Resilience with dedicated mental health support.^46^^,^^47^^,^^50^

If sustained by an environment where Policy, Funding, Leadership, Education, and Technology^50^^,^^54^ are used as strategic levers; this values-based model could offer a path to retaining practitioners in rural areas.

### Limitations of this study

This systematic review synthesises the latest global evidence on physician stress, rural workforce attrition and contributing/mitigating factors across multiple health system contexts. Despite heterogeneity in study designs and healthcare settings, recurring patterns were identified across moderate- and high-quality studies, suggesting consistency in reported outcomes.

Several limitations remain. The 5-year search window and exclusion of COVID–19–specific studies may limit the current understanding of post-pandemic realities. The papers identified were geographically skewed, with a predominance of studies from China. This limited generalizability, as many low-income and non-English-speaking rural health systems remain underrepresented. Nevertheless, all studies were appraised using a validated tool (QuADS), and theme confidence was guided by GRADE-CERQual principles (Table 2).^77^ Still, caution is warranted in cases of small sample sizes or limited analytical transparency.

**Table 2:** Summary of themes and associated confidence ratings. Confidence ratings are based on an adapted approach informed by GRADE-CERQual principles.

	Section A: outcomes of stress				
Theme	No. of studies	High-quality studies	Moderate-high-quality studies	Moderate-quality studies	Overall confidence
**Burnout and emotional exhaustion**	13	(Hansen et al., 2021), (Lesperance et al., 2022), (Clough et al., 2020), (Harry et al., 2024)	(Latham et al., 2025)	(Zhang et al., 2021), (Zhao et al., 2021), (Zhu et al., 2023), (Fitzpatrick et al., 2020), (Ward et al., 2021), (Hain et al., 2021), (Purbrick et al., 2024)	Moderate-to-High
**General psychological distress**	6			(Ji et al., 2023), (Fitzpatrick et al., 2020), (Kuroda et al., 2022), (Hain et al., 2021), (Purbrick et al., 2024).	Moderate
**Retention and disengagement**	11	(Wang et al., 2023), (Yan & Sun, 2022), (Hadley, 2024), (Jolicoeur et al., 2022), (Eaton-Hart et al., 2022)	(Latham et al., 2025)	(Zhang et al., 2020), (Islam et al., 2024), (Naher et al., 2022), (Hain et al., 2021), (Purbrick et al., 2024).	High
**Section B: contributing factors (sources of stress or support)**					
**Structural, organisational, and professional stressors**	24	(Wang et al., 2023), (Yan & Sun, 2022), (Hadley, 2024), Hansen et al., 2021), (Jolicoeur et al., 2022), (Lesperance et al., 2022), (Clough et al., 2020), (Eaton-Hart et al., 2022), (Harry et al., 2024)	(Latham et al., 2025)	(Ji et al., 2023), (Zhang et al., 2021), (Zhang et al., 2020), (Zhao et al., 2021),(Zhu et al., 2023),(Fitzpatrick et al., 2020), (Ward et al., 2021), (Kuroda et al., 2022), (Islam et al., 2024), (Naher et al., 2022), (Hain et al., 2021), (Purbrick et al., 2024).	High
**Personal and interpersonal Stressors**	15	(Yan & Sun, 2022), (Hadley, 2024), (Jolicoeur et al., 2022), (Lesperance et al., 2022), (Harry et al., 2024)	(Latham et al., 2025)	(Zhang et al., 2021), (Zhao et al., 2021), (Ward et al., 2021), (Islam et al., 2024), (Naher et al., 2022), (Hain et al., 2021), (Purbrick et al., 2024).	High
**Community and contextual factors**	9	(Hadley, 2024), (Jolicoeur et al., 2022), (Lesperance et al., 2022)	(Latham et al., 2025)	(Zhu et al., 2023), (Kuroda et al., 2022), (Naher et al., 2022).	High
**Mitigating and protective factors**	11	(Hansen et al., 2021), (Jolicoeur et al., 2022), (Lesperance et al., 2022), (Harry et al., 2024)	(Latham et al., 2025)	(Zhang et al., 2020), (Zhu et al., 2023), (Fitzpatrick et al., 2020), (Hain et al., 2021), (Purbrick et al., 2024).	High

Future research should extend to underrepresented regions, include non-English literature, and employ longitudinal designs to better understand the impacts of interventions on physician retention. Additionally, a deeper investigation is required into how cultural, economic, and health system differences influence the generalizability of the proposed Disruptive Retention Framework. This will support globally relevant, context-specific policy development.

## Supplementary Material

Supplemental_Data_S1_fdag011

Supplemental_Data_S2_fdag011

Supplemental_Data_S3_fdag011

Supplemental_Data_S4_fdag011

Supplemental_Data_S5_fdag011

## Data Availability

Data sharing does not apply to this article as no new data were created or analysed in this study.

## Notes

**Joëlle V.F. Coumans**, Senior Lecturer

**Ilona Ciller**, Lecturer
