# Supplementary figures and images for: Stress and retention challenges among rural and regional physicians: a mixed-methods systematic review and framework for action
Source: J Public Health (Oxf). 2026 Feb 15;48(2):572–81. doi: 10.1093/pubmed/fdag011 (PMC13223598; doi:10.1093/pubmed/fdag011)

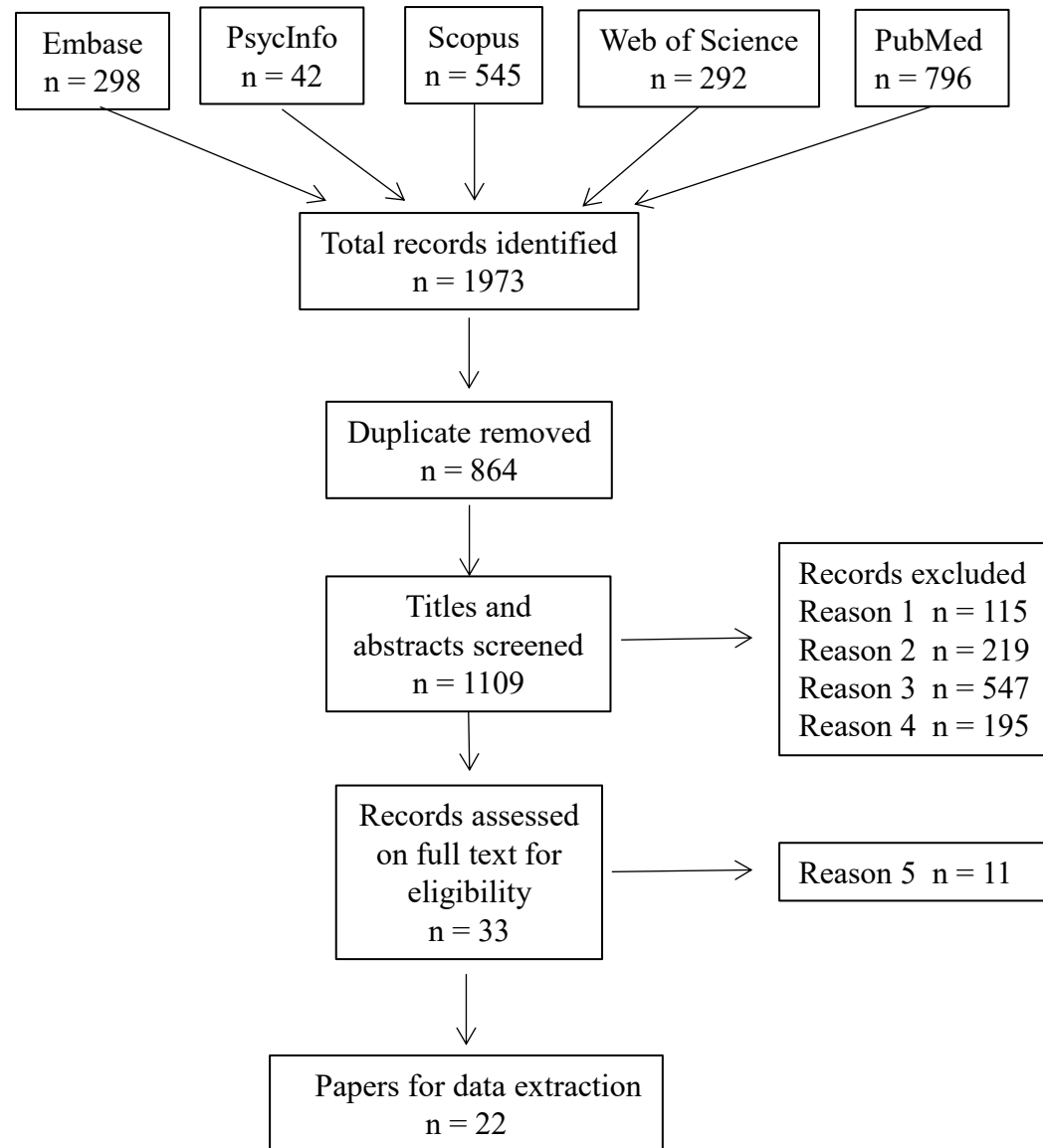

Supplemental Data S1: PRISMA Flow Diagram for the systematic review process

Supplement: Supplemental_Data_S1_fdag011 [file supplemental_data_s1_fdag011.pdf]
