# Supplementary material for: Stress and retention challenges among rural and regional physicians: a mixed-methods systematic review and framework for action
Source: J Public Health (Oxf). 2026 Feb 15;48(2):572–81. doi: 10.1093/pubmed/fdag011 (PMC13223598; doi:10.1093/pubmed/fdag011)
Supplement: Supplemental_Data_S2_fdag011 [file supplemental_data_s2_fdag011.docx]

| **Healthcare workers** | **Area** | **Sleep disturbance** | **Stress** | **Anxiety** | **Depression** |
| --- | --- | --- | --- | --- | --- |
| 1. “general practice" 2. “primary care physician*" 3. "family practice" 4. "family physician*" 5. "primary health care" 6. doctor* 7. medicin* 8. “medical practitioner*” 9. “medical provider” 10. physician* 11. “general practitioner*” 12. “GP” 13. “GPs” 14. OR/ #1 - #13 in title | 1. rural 2. regional 3. remote 4. provinc* 5. OR/#15 - #18 in title and authors' keywords | 1. insomnia 2. “sleep disorder*” 3. “sleep problem*” 4. fatigue 5. OR/ #20 - #23 in title, abstract and author keywords | 1. stress* 2. burnout 3. “burn out” 4. workload 5. "work-load" 6. exhaustion 7. pressure* 8. burden 9. OR / #25 - #32 in title, abstract and author keywords | 1. anxiety 2. anxious* 3. distress 4. OR/ #34 - # 37 in title, abstract and author keywords | 1. depress* 2. “mental health” 3. “psychological problem*” 4. “psychological disorder*” 5. “post trauma*” 6. post-trauma* 7. “psychiatric morbidit*” 8. OR/#38 - #44 in title, abstract and author keywords |
| Combined search | 1. #24 OR #33 OR #37 OR #45 2. #14 AND #19 3. #46 AND #47 | | | | |
| Restricted #48 to | English  Last five years (January 2020 to January 2025) | | | | |

Supplemental Data, S2: Search strategies across the different databases (Embase, PsycInfo, Scopus, Web of Sciences and PubMed).
