# Supplementary material for: Stress and retention challenges among rural and regional physicians: a mixed-methods systematic review and framework for action
Source: J Public Health (Oxf). 2026 Feb 15;48(2):572–81. doi: 10.1093/pubmed/fdag011 (PMC13223598; doi:10.1093/pubmed/fdag011)
Supplement: Supplemental_Data_S3_fdag011 [file supplemental_data_s3_fdag011.docx]

**Supplemental Data, S3:** Summary of papers included in this systematic review.

| **Authors, study location, and funding** | **Aims** | **Participants demographics** | **Research design (1) and analysis methods (2)** | **Main findings** | **Implications/Recommendations** | **Quality assessment** |
| --- | --- | --- | --- | --- | --- | --- |
| (Ji et al., 2023)  China  Supported by the National Natural Science Foundation of China, the Social Science Foundation of Jiangsu Province, and the Jiangsu Overseas Visiting Scholar Program for  University Prominent Young & Middle-aged Teachers and Presidents (2017).and Presidents (2017). | Explore the role of depressive symptoms and effort-reward imbalance (ERI) in the relationship between long working hours and presenteeism among village doctors. | Village doctors (n=705, mean age, 45.25 ± 12.36, 40.28% male) | Cross-sectional study in three districts (Tongzhou district  and Haimen District in Nantong City, Dafeng District in Yancheng City) in Jiangsu Province, eastern China.  Mean weekly working hours ((mean working hours per day) × (working days)) rounded up to the nearest hour.  Effort-Reward Imbalance: ERI questionnaire  Depressive symptoms: GHQ-12 questionnaire  Presenteeism: SPS-6 | 45.11% worked more than 55 h per week.  55.89% were exposed to ERI.  40.85% have depressive symptoms.  Positive correlation between working hours and ERI with both depressive symptoms and presenteeism (P<0.001).  Depressive symptoms were also positively correlated with presenteeism (P < 0.001).  Working longer hours significantly predicted higher depressive symptoms (P < 0.001), which, in turn, was significantly associated with a high presenteeism score (P < 0.001).  ERI strengthened the detrimental effect of long working hours on depressive symptoms. | - Regularly assess the psychological well-being of village doctors. - Reduce excessive workloads and long working hours to mitigate presenteeism. - Foster a supportive environment by reducing bureaucracy and improving job security. - Improve compensation schemes and address physicians' psychosocial needs. | Moderate  The study has a clear goal, appropriate methods, including consideration of ethical issues, and relevant data collection and analysis that enable practical recommendations to be made. The study's limitations include the absence of information regarding the handling of missing data, non-participation, and a sensitivity analysis. |
| (Wang et al., 2023)  China  Funded by the National Natural Science Foundation of China, “Youth Innovation and Technology Program” Project of Colleges and Universities in Shandong Province, the Humanities and Social Science Foundation of the Ministry of Education of China, and the Key Research and Development Program of Shandong Province. | Explores how the surface acting of rural family doctors affects turnover intention through emotional exhaustion and investigates what role occupational commitment plays in this relationship. | Family doctors in rural China (n = 953)  The mean age is 46.65 years, with 62.85% being male. | Cross-sectional study.  The questionnaire covered the scales of surface acting (five items), emotional exhaustion (EE) (five items), occupational commitment (six items), and turnover intention (three items). | Surface acting predicted turnover intention (β = 0.481, P < 0.001), and EE (β = 0.532, P < 0.001)  EE predicted turnover intention (β = 0.403, P < 0.001).  EE has a partial mediating role because the direct effect of surface acting on turnover intention (β = 0.267, P < 0.001).  EE X occupational commitment predicted turnover intention (β = − 0.065, P < 0.01). | - Provide emotion management training to reduce physician turnover. - Promote engagement strategies over surface acting to improve empathy and reduce burnout. - Improve public understanding of the family doctor system to enhance patient cooperation. - Reinforce occupational commitment and provide external support to mitigate burnout and turnover. - Conduct longitudinal research on individual and organisational factors influencing turnover. | High  The use of validated scales, a high response rate, and robust statistical methods contributes to the study's strength. The practical implications for health policy and interventions are well-described. |
| (Yan & Sun, 2022)  China  Funded by the Project for Philosophy and Social Science Research in Colleges and Universities in Jiangsu Province | Investigate how we could anticipate the loss of rural doctors to facilitate their retention in advance | Rural physicians (n = 722, 47.2% Male, 89.6% were 40 years and older) from:  Northern Jiangsu (45.6%) (per capita GDP = 75,551 RMB).  Central Jiangsu (26.0%) (per capita GDP = 123,551 RMB).  Southern Jiangsu (28.4%) (per capita GDP = 167,995 RMB). | Cross-sectional survey with a reliable multi-stage stratified sampling method.  Questionnaires include:   - demographic information, - work conditions. - monthly expenditure items.   A key question was asked to all participants to evaluate the anticipated rural retention “Do you intend to work at your current workplace until retirement?” | Anticipated retention rates:  - Northern Jiangsu (64.4%)  - Central Jiangsu (75.5%)  - Southern Jiangsu (80.0%)  Factors associated with rural doctors' anticipated retention.  *Protective factors:*   - Areas:   - Central Jiangsu (OR = 0.501, p = 0.003)   - Southern Jiangsu (OR = 0.475, p = 0.002)   Higher monthly income:  o ~3000 RMB (OR = 0.584, P = 0.03)  o ~6000 RMB (OR = 0.255, P= 0.001)  - Advanced rank among counterparts (OR = 0.507, P = 0.036)  *Detrimental factors*:  Socialisation with others (OR = 1.856, P = 0.002)  Working hours per week (50~) (OR = 2.076, P = 0.007)  Outpatient service (OR = 1.991, P=0.01)  File work (OR = 1.544, P = 0.025) | - Strengthen economic incentives and reduce workloads to improve retention. - Focus rural policy on improving income and infrastructure. - Implement a comprehensive, multi-level government strategy for rural healthcare. - Provide regular postgraduate education to enhance quality and retention. - Integrate recruitment and retention strategies to address workforce shortages. - Apply research findings to develop targeted retention policies and practices. | Moderate  The study benefits from a large sample size and the use of appropriate and rigorous statistical methods to identify factors associated with retention. However, its overall quality is limited by a convenience sampling strategy (distribution via WeChat groups) that carries a high risk of selection bias, threatening the generalisability of the findings. |
| (Zhang et al., 2020)  China  Funded by the Teacher Support Fund of Jining Medical University. | Evaluate the influence of job satisfaction, resilience, and work engagement on the village doctors’ turnover intention. | Village doctors (n = 2693, 64.5% male, average age 44.6 ± 7.3 years) | Cross-sectional study  Quantitative study using a self-administered questionnaire including:  Demographic data  Medical Staff Job Descriptive Index  Utrecht Work Engagement Scale  Connor-Davidson Elasticity Scale (CD-RISC) for resilience.  Chinese Turnover Intention Scale | Scores for:   - job satisfaction (32.48 ± 8.93), - resilience (74.01 ± 17.06), - work engagement (66.14 ± 20.26) - turnover intention (12.16 ± 6.09), with 46.9% having high turnover intention.   Negative correlation between turnover intention and job satisfaction, resilience, and work engagement. | - Increase physician income, establish clear career pathways, and provide social security. - Reduce workloads and support positive coping strategies to build resilience. - Prioritise improving professional conditions, such as income, training, and promotion. - Utilise qualitative research to comprehend resilience and identify effective protective measures. | Moderate  The study is methodologically strong, with a clear aim, a large representative sample, and a sophisticated analytical approach (SEM) that yields practical recommendations. Its overall quality is tempered by a significant and unaddressed risk of self-report bias, as all predictor and outcome variables (job satisfaction, resilience, turnover intention) were collected from the same source at the same time, which may inflate the observed relationships, warranting a moderate rating. |
| (Zhang et al., 2021)  China  Funded by Jining Medical University 2018 University-level Education and Teaching Research Project, Research on the Influence of the Application of Situational and Inquiry Integrated Teaching Mode in the Teaching of Social Medicine on the Independent Learning Ability of Clinical Undergraduates. | Evaluate the effects of personality, work engagement and alexithymia on burnout of village doctors. | Village doctors (n = 2684, mean age 44.64 ± 7.248 years, 64.4% male).  University or above (2.6%)  Junior College (24.4%)  Technical secondary school (68.2%)  High school education or below (3.4%)  Missing (1.4%)  Rural general practitioner (13%)  Rural assistant physician (17.2%)  Chinese medicine assistant physician (7.5%)  Unqualified (62.4%)  Missing (4.1%) | Cross-sectional study  Self-completed questionnaire:   - demographic characteristics - job characteristics. - Burnout (MBI-GS) - personality (BFI). - Alexithymia (TAS-20)   work engagement (UWES) | 69.9 % worked > 60 hours/week.  46.5% earn less than 2000 yuan a month.  Burnout  65.2% were diagnosed with burnout.   - severe emotional exhaustion (30.1%%) - moderate emotional exhaustion (24.2%) - low emotional exhaustion. (45.7%)   - severe low personal achievement (48.3%)  - moderate low personal achievement (13.3%)  - low personal achievement (38.4%)  - severe depersonalization (22.2%)  - moderate depersonalization (11.7%)  - Low depersonalization burnout. (66.0%)  Alexithymia (25.3%)  Personality had a positive effect on work engagement (p < 0.001) and an adverse effect on alexithymia (p < 0.001) and burnout (p < 0.001).  Work engagement had an adverse effect on burnout (p < 0.001).  Alexithymia had a positive effect on burnout (p < 0.001).  In the path between personality and burnout, both work engagement 95% CI:(‒ 0.17)-(‒ 0.08), and alexithymia 95% CI: (‒ 0.36)-( ‒ 0.09), have significant mediating effects.  These results strongly confirm that personality, alexithymia, and work engagement are early and powerful predictors of burnout. | - Integrate personality and psychological assessments into medical training and hiring. - Screen for and address alexithymia as an early burnout indicator. - Use personality assessments to inform physician job assignments. - Provide stress and emotion management training for physicians. - Conduct further research on burnout causes and effective interventions. | Moderate  The study is methodologically strong, with a clear aim, a large representative sample, and a sophisticated analytical approach (SEM) that yields practical recommendations. The overall quality is tempered by an unaddressed risk of self-report bias, as all predictor and outcome variables (personality, alexithymia, burnout) were collected from the same source at the same time. This may inflate the observed relationships. |
| (Zhao et al., 2021)  China  Funded by the National Commission of Health of China | Investigate the prevalence and influential factors of burnout among village doctors. | Village doctors (n = 1248, 71.07% male, 71.72% < 50 years old) | Cross-sectional study  Self-administered questionnaire including:   - Demographic data - Job-related variables   MBI-HSS | 67.31% with ≤ high school and 5.69% with bachelor’s degree.  87.51% > average self-rated health.  93.99% no vacation leave  90.22% > average doctor-patient relationship.  Burnout (23.6%)  Emotional exhaustion (29.61 ± 16.19)  Depersonalization (4.84 ± 6.74)  Personal accomplishment (36.03 ± 10.16).  Factors related to burnout.  Male (p < 0.01)  Poorer health (p < 0.01)  Lower income (p < 0.05)  Poorer doctor-patient (p < 0.001) | - Increase rural physician income and healthcare investment. - Implement individual-level stress reduction strategies. - Invest in improving rural work environments. - Address staffing shortages through training and recruitment (e.g., retired doctors). - Promote temporary rural placements for urban physicians. - Provide targeted support during public health crises. | Moderate  The study has a clear aim and uses appropriate, well-described methods for measurement and analysis. However, its trustworthiness and generalisability are limited by methodological issues. (1) the use of a convenience sample of doctors attending a training program introduces a high risk of selection bias, making it difficult to generalise the findings. (2) A cross-sectional design can only establish association, not causality.  (3) A reliance on self-report data creates a potential for recall and social desirability biases. |
| (Zhu et al., 2023)  China  No funding | Delineate the workload and identify predictors of burnout for physicians at village clinics in rural regions (the southwestern province of  Guizhou, China). | Village doctors (n = 79, 45.57% male, Age, mean ± SD (38.70 ± 8.13 years))  Professor village physician (1.27%)  Village physician in charge (2.53%)  Assistant village physician (29.11%)  Other (24.05%)  No title (43.04%) | Cross‐sectional survey using an online questionnaire including:   - Demographic data - Work characteristics.   OLBI (burnout) | 45.57% junior school and 12.66% bachelor’s degree.  Work hours/week:   - 40-49 (24.05%) - 50-74 (56.96%)   Workload composition  Basic public health service dominated (87.34%)  Performance bonus (62.03%)  Number of received support special consultations by physicians from higher‐level facilities, sharing information about testing and imaging within the alliance, and telemedicine) (per year [time (s)] 1-5 (62.03%).  Burnout, mean± SD (38.09% ± 4.55)  Significant positive association:   - Fewer work years (6-10 years, p = 0.041; 11-20 years p = 0.034) - Too much farm work (p = 0.028)   Significant negative association  Sharing more medical services (p = 0.007)  Support from higher‐level facilities (>5) (p = 0.016) | - Strengthen primary care capacity and public health services. - Empower physicians to improve satisfaction and system efficacy. - Resolve rural understaffing and resource shortages. - Broaden medical alliance evaluations beyond efficiency metrics. - Increase leadership and government support for village physicians. - Develop tailored rural strategies (e.g., online training, local recruitment). - Innovate and diversify physician payment models. - Expand research on the causes and impacts of village physician burnout. | Moderate  The study is methodologically sound with clear findings and appropriate use of statistical methods. There are limitations related to a small convenience sample, a lack of detail regarding handling missing data, and a cross-sectional design that prevents causal inference |
| (Hadley, 2024)  Canada  Funded in part by the Canada Graduate Scholarship - Master’s program and the University of Alberta graduate scholarships. | Explore why South African educated physicians make decisions about migrating to or from rural Alberta (e.g., situates rural doctors’ decisions to remain in rural Alberta in the context of their broader migration trajectory), and to understand what role professional mobility plays in South African physicians’ geographic mobility (e.g., importance of professional migration with the ability to realise professional goals). | South African physicians (n = 29) who received education in their own country, immigrated to Canada, and practised as general practitioners or family medicine specialists in rural Alberta for at least six months.  93.1 % within the age range of 30–59-year-old, 65.5% male.  Married/Common Law (62.1%), and 27.6% with children at migration | Semi-structured qualitative  Interviews with open-thematic coding guided by abductive grounded theory using NVivo software. | Migration decisions:  A perceived general lack of professional opportunities coincided with an experienced lifestyle time window when migration was possible.  The aspiration to achieve prestige and the opportunity to pursue specialisation (short-term stay).  Friends and family in the province.  Community criteria, such as amenities.  Absence of Canadian-educated physicians in rural family practice.  Access to subspecialist family practices that allow better working conditions.  Stay in rural practices because:  Their migration aspirations for professional mobility were met. Hadley states this is the key: they stay when the rural job provides unique pathways for upward mobility (e.g., focused clinical or administrative roles) that they couldn't get elsewhere.  They were satisfied with the work, and the opportunities still outranked those in South Africa.  Their family was content, and they felt an attachment to the community.  Leave rural practices because:  The professional aspiration was frustrated. This could be through:  Deskilling: Being limited to lower acuity work and losing procedural skills.  Blocked Pathways: Finding that subspecialist training came with restrictive "return of service" agreements.  Lack of Mobility: Realising that even moving to an urban centre would result in a "second deskilling" into a more limited clinic-based role.  The personal cost became too high. This was driven by:  Burnout and feeling "trapped in a practice without reprieve."  The need to prioritise personal wellness over income.  Family unhappiness or a partner's needs dictating a move.  Experiences of racism for physicians of colour. | - Revise international medical graduates (IMG) migration and retention policies to be more nuanced and effective in addressing rural shortages. - Focus retention strategies on professional factors, such as career aspirations and mobility, over community aspects. - Conduct further research on IMG destination choices, regulatory impacts, and the experiences of diverse and non-family medicine specialists. | High  The paper's methodology, ethical considerations, and data analysis are well described all of which is further enhanced by a self-critical discussion of its limitations. The non-probability sample limits statistical generalisability, but this is appropriate for the qualitative exploratory aims. |
| (Hansen et al., 2021)  Canada  Support was provided by the Canadian Institutes of Health Research Strategy for Patient-Oriented Research, PICHIN Operations and Management grant. | Examine the relationships between structural factors in the health system, including governance, organisation, and work environment-related issues, and physician burnout in three northern territories in Canada – Nunavut, NWT, and Yukon. | Physicians (n= 57) from any specialty, Yukon (n=10), NWT (n=3), Nunavut (n=44), 28% male.  Primary care (n=38)  Medical specialty (n=8)  Surgery (n=11) | Mixed methods study that combined a cross-sectional survey (adapted from MBI) and in-depth qualitative interviews. | MBI profile   - Most participants (n = 35) fit into the ‘engaged’ profile, and relatively few into ‘burnout’ (n = 2) or ‘disengaged’ (n = 0). - Inverse correlation between the ‘engaged’ MBI profile and use of electronic medical records (p < .05), inadequate financial remuneration (p < .05) and cross-cultural issues (p <0.05). - Direct association between the ‘engaged” profile and intrinsic love of work (p < .05) and support from coworkers (p < .05).   Contributing factors  - Lack of influence in the health system due to administrative structures, lack of transparency, and lack of direct involvement in health policymaking.  - Systemic failures (lack of education about Indigenous cultures, history, and experiences; and minimal Indigenous representation in the healthcare workforce as contributors) to provide culturally safe care and meet the needs of Indigenous patients.  - Inability to provide continuous care to their patients, due to the ratio of providers to the population size and distribution.  - Quantity of work (rooming patients, entering labs into the electronic medical record, and cumbersome paperwork processes) assigned to them that falls outside their scope of practice.  - High rates of physician turnover and reliance on locum providers that require significant orientation and training investments, and are seen as providing lower quality, less efficient care.  - Lack of services such as physician health programs, continuing education, and financial planning.  Mitigating factors  - Relationships physicians have with their colleagues and communities.  - Ability to spend time on the land, in nature, allows physicians to disconnect from their work-life, to build deep and meaningful relationships with their community, and to witness Indigenous cultures and gain context for what health means to the people they serve.  Double-edged swords  - Amount of support they received from both family physicians and specialists, however, if they felt thrust into situations beyond their comfort level with inadequate support, that became stressful.  - Lack of boundaries between work and personal life in the doctors/patient’s relationship.  - Vacation time is a protective factor but also interferes with relationship building and continuity of care. | - Address systemic and cross-cultural drivers of burnout. - Increase physician influence on policy and administration. - Enhance Indigenous cultural safety within the health system. - Prioritise physician retention over recruitment. - Manage workloads and resolve understaffing. - Strengthen community integration and support. - Research structural racism and communication as burnout drivers. | High  The study has an appropriate explanatory sequential mixed-methods design to address the research question; data collection, analysis, and integration of mixed-methods data explains and adds crucial depth to the survey results. While the study is constrained by a low survey response rate, this limitation is discussed by the authors. |
| (Jolicoeur et al., 2022)  Canada  Supported by NOSM Summer Student Research  Grant, Northern Ontario Academic Medicine Association  Academic Funding Plan Innovation Fund 2018. | Explore influential factors leading to physician turnover in designated Rural Northern Physician Group Agreement (RNPGA) communities in Northern Ontario, as well as physicians’ perceptions of the RNPGA contract and effects of the Northern Ontario School of Medicine (NOSM) on physician retention in these communities. | Physicians (n=12 from 11 different communities, 9 male, years spent in the community of practice ranged  from one to 27 | Interviews and thematic analysis. | Personal challenges   - Partner’s lack of career opportunities led to partner and personal dissatisfaction with life in the community. - Desire to be near extended family and immediate family member’s illness. - Uncertainties in their future life plans. *It’s pretty easy for a rural community to turn from sort of supportive and nurturing to kind of oppressive and boring.*’ - A sense of feeling isolated and unaccepted, leading to trouble finding meaningful community relationships   Professional support challenges   - Lack of human and non‑human resources, with the lack of human resources contributing to a sense of isolation. - Poor team dynamics - Lack of flexibility of the RNPGA contract.   Professional experience challenges   - Burnout and work‑induced stress. - Short of physicians, never meeting their quota associated with patients having medically complex and vulnerable. - Personal responsibility for health outcomes   Rural community lifestyle challenges   - Lack of community opportunities and amenities (dependable childcare, education, and sports‑related opportunities) - Enhanced patient expectations of care, dual roles, and lack of personal privacy. Some dilemmas in caring for friends and family members. - Some participants maintained permanent addresses in nearby urban settings and would commute to work.   Northern Ontario school of medicine  NOSM’s initiatives may keep physicians in the North, but not in the rural North.  Frustration being unable to teach NOSM residents or recruit new graduates.  Difficulty engaging in NOSM‑related opportunities was attributed to geography, time constraints, workload, and a perceived lack of relationship between NOSM and the  practice community.  Sense of rural generalist practice being a ‘double‑edged sword. “…*some of the things that one enjoys about a rural community are also part of the challenges.”* | - Use physician turnover data to inform community recruitment and retention policies. - Revise physician contracts to offer part-time work, increase funding, and incentivize long-term integration and wellness. - Implement mandatory rural postgraduate placements and improve supporting infrastructure. - Focus on bridging the gap between physicians and communities to improve the balance of rural practice. | High  The study has a precise aim, employs appropriate qualitative methods, and the thematic analysis ensures a comprehensive understanding of the research issue. There is, however, a limitation in the lack of detailed discussion on the researchers' potential biases and ethical considerations. |
| (Lesperance et al., 2022)  Canada  Supported by college of Family Physicians of Canada, Janus Research Grant. Newfoundland and Labrador Medical Association Wellness Grant. | Understand how Rural family physicians (RFPs) in Canada define and use resilience to maintain their generalist scope of practice. | Purposive and theoretical sampling (n = 14).  Age range: 35 -75 years, 50% male and 1-30 years of experience in rural practice | Semi‑structured, in‑depth interviews based on grounded theory, including questions on resilience, challenging experiences, health and well‑being, coping strategies and background.  Themes analysis based on three coding strategies of grounded theory (open, axial and selective coding). | Based on the definition of ‘*resilience*’ in the workplace, RFPs mentioned factors ranging from the ability to bounce back and survive, to creating strategies for getting through stressful circumstances  **Factors associated with**:  Powerlessness:   - administrative systems impacting the getting a deep understanding of patients’ issues and needs. As a result, they ‘*choose to accept it or choose to walk away*’. - Participants described burnout, compassion fatigue, and emotional exhaustion related to rural workload, moral outrage, and systemic pressures (e.g., ‘I am closing my practice. I’m burnt... you just cannot do it anymore’).” - lack of equipment in remote areas, principally in Indigenous communities. - additional often unpaid duties (e.g., mentorship, teaching, and committee membership).   Work/life balance:   - engaging in different challenges in the workplace. - manage a broad array of duties and night shifts due to a lack of staff and supplies. - blurred zone between life and their job. - lifetime trauma.   Colleagues:  *Supportive*:   - talking to and debriefing - No judgment on those asking questions or looking for assistance.   *Straining*   - source of stigmatisation and exclusion. - questioning medical decision‑making the professional review. - victim of workplace violence or ‘toxic environment’.   Living under a microscope  small community and being known by almost everyone.  stigmatisation of counselling services.  Compassion fatigue or empathy.   - limited skills in emotional regulation and boundary setting related to a high degree of clinical empathy.   Mitigating factors mentioned:  Personal:   - mindfulness-based meditation - living life outside of work - healthy lifestyle. - participation in counselling services.   Community   - strong support network - debriefing - having a mentor - instilling a culture of ‘*no one can know everything*’.   Organisation   - involvement in major decisions. - access to expert counsellors. - resilience-related classes.   remuneration for a mentor | - Promote individual and organisational strategies to build resilience. - Empower rural physicians by including them in decision-making processes. - Build strong colleague support networks and positive team dynamics. - Utilize virtual care and telemental health services for support. - Tailor training and organizational programs to address unique rural challenges. - Implement the Truth and Reconciliation Commission's Calls to Action. - Conduct further research on systemic factors that enhance rural resilience. | High  The paper provides a clear aim, appropriate methodology, thorough data collection and analysis, and valuable findings relevant to the field of rural physician resilience. The paper could improve by providing more detailed discussions on researcher-participant relationships and ethical considerations. |
| (Clough et al., 2020)  Australia  No funding reported | Compare stress, burnout, stressors, and protective factors among regional‐ and metropolitan‐based Australian medical doctors. | Quantitative sample  Doctors (n = 252, mean age 37.47 ± 9.31), 75.0% female, 15.9% male, 9.1% nonresponse).  73.4 % specialists with:  - general practice (37.3%)  - emergency medicine (6.3%)  - psychiatry (6.3%)  - paediatrics (6.0%).  Area   - major cities (59.5%), - inner regional (16.3%), - outer regional (6.7%), - remote (2.8%), - very remote (1.6%)   Qualitative sample  Regional (n = 11, mean age 39.01 ± 8.57, 9 females)  Metropolitan (n = 9, mean age 39.78 ± 9.60, 5 females) | Mixed methods design  Stress and burnout.  DASS‐21, CBI  Stressors  COPSOQ‐II  Semi-structured interview with open‐ended questions | Stress and burnout.  No statistically significant difference in stress, personal, work-related, or client-related burnout between metropolitan, regional, or remote.  Stressors in the Work environment.  There is a significant variation ↑ between regional and metropolitan areas.  Quantitative demands ↑ in regional vs remote.  Influence ↑ in regional vs metropolitan and remote.  Meaning of work ↑ in regional vs metropolitan and remote.  Gossip/slander ↑ in metropolitan vs regional.  Quarrels/conflicts ↑ in remote vs metropolitan and regional areas.  Unpleasant teasing ↑ in remote vs metropolitan and regional areas.  Threats of violence ↑ in remote vs metropolitan and regional areas.  Physical violence ↑ in metropolitan vs regional.  Qualitative themes of stressors and protective factors  Three major themes: system‐level factors, clinical environment factors, and individual factors and personal responses.  Specific to regional area  *System Level Factors:*   - Limited resources in terms of a lack of access to specialist and allied health services, as well as access to other doctors to reduce working hours or take leave, are reported by almost all regional doctors.   *Clinical environment factors*   - Clinical interest was reported across samples as a protective factor and included an individual’s intellectual curiosity, contained variety, and allowed the individual to develop skills better in regional practice, as there is an opportunity to engage in a greater variety of work due to the limited resources. - Lack of Support (supervisors, management, or other health professionals) and bullying among all samples, but predominantly in regional (27.27%) vs metropolitan (45.45%). - Autonomy and boundaries, both stressors and protective factors occurring on a continuum, included control over working hours, patient load, or periods of rest (whether within the workplace or after working hours), which regional doctors and stakeholders reported.   *Individual Factors and Personal Responses*  Perceived Community and connection identified in regional doctors regarding dual relationships and personal involvement with patients as stress and buffer factors. | - Future interventions should target identified stressors and protective factors.  - Address work-family conflict and work-life boundaries.  - Provide greater resources for regional doctors.  - Develop personal traits like resilience.  - Offer protected time for preventative programs.  - Promote flexible working hours and role autonomy.  - Improve system interaction and communication. | High  The use of a mixed-method approach in this study provides effective integration of findings while adhering to high-quality standards in both quantitative and qualitative methods. This is unfortunately limited by the use of a non-probability, self-selected sample, a limitation the authors acknowledge. |
| (Fitzpatrick et al., 2020)  Australia  No funding reported | Assess the associations between individual, community, and hospital factors with emotional exhaustion (EE) among rural Australian doctors in training (DITs) and to apply criteria from an international standard that measures sustainable employability in organisations. | Rural DITs (n = 70, 65 fully completing, 60% male).  Intern (31.4%)  Resident (21.4%)  Senior medical officer (10%)  Career medical officer (1.4%),  Registrar (34.3%)  Advanced trainee (1.4). | Cross-sectional study  Factors analysed:   - Rural background, prior   rural university placements, commute time and cost of living.  Occupational health factors.   - presenteeism and absenteeism questions. - Three elements of the WAI. - Nine-item EE portion of the MBI. - Four items of the Effort–Reward Imbalance Scale. - Work-related sleep disturbance: ‘I can’t sleep well because I think about work.   Personal health factors  Kessler-6 scale (K6)  Hospital-level metrics  The (ISO) guidelines on sustainable employability management for organisations. | Both interns and other trainees had lower rates of high EE than the national data, including rural and urban DITs.  Individual and lifestyle factors associated with EE.  Univariate logistic regressions predicting high EE among DITs with:   - Ability to cope with the mental work demands (OR8.27; 95% CI 1.69–40.42). - Sleep disturbances (OR 6.19; 95% CI 1.72–22.24), - Higher K6 scores (OR 1.70; 95% CI 1.29–2.25). - Hours worked on-call (OR 1.05; 95% CI 1.01 – 1.09) - Increased presenteeism (OR 1.29; 95% CI 1.05–1.58).   Not significant association with EE:   - Cost of living. - Commute time.   Organisational factors associated with EE  All variables related to the ISO guidelines of workplace factors had a significant association with EE.  Support networks in the hospital (p<0.022)  The hospital encourages doctors:   - to have a healthy lifestyle balanced with the physical demands of their job, and provide support mechanisms to achieve this (p<0.010) - to enhance their mental health balanced with the cognitive demands of their job, and provide support mechanisms to achieve this (p<0.024)   The hospital allows flexibility on working hours and schedules where possible to accommodate the doctor’s personal life needs (p<0.046)  The hospital promotes the overall well-being of doctors as a priority (p<0.005).  Univariate logistic regressions predicting high EE among DITs with:  Unsatisfied with support networks in their hospital (OR 3.323; 95% CI 1.191–9.273).  Those who disagree that the hospital promotes the overall well-being of doctors as a priority (OR 8.800; 95% CI 1.920–40.336). | Organizational & Practice Recommendations:   - Adopt an organization-focused approach to prevent emotional exhaustion. - Prioritise the creation of supportive environments for doctors-in-training. - Implement the international standard for sustainable employability to assess and improve physician well-being.   Policy Recommendations:   - Develop and tailor interventions specifically for rural doctors-in-training to address emotional exhaustion.   Future Research:   - Investigate the effectiveness of specific hospital-led strategies designed to improve the well-being of junior doctors. | Moderate  Thorough reporting of methods and results with clear definitions of variables and outcomes. The discussion includes limitations and generalisability.  The small convenience sample with a low response rate introduces a risk of selection bias. |
| (Eaton-Hart et al., 2022)  Scotland  No funding reported | Compare the working lives and intentions to reduce work participation of rural GPs and GPs working elsewhere in Scotland. | GPs in rural (n = 347, 41.8% males).  GPs in non-rural (n = 2085, 41.6% males).  No significant differences in the age, gender or proportion of GPs who are GP practice partners. | Cross-sectional analysis  Quantitative analysis of survey data of four domains of working lives: job satisfaction, job stressors, positive and negative job attributes, and four intentions to reduce work participation: reducing working hours, working abroad, leaving direct patient care, and leaving medical work entirely. | Working lives  Rural GPs reported significantly higher mean job satisfaction than non-rural GPs because of the amount of free choice (p=0.008), recognition (p<0.001), job responsibility (p=0.018), remuneration (p=0.002), variation (p<0.001) and job opportunities (p<0.001).  Rural GPs reported significantly lower mean job stressors relating to patient demands (p<0.001), time (p<0.001), administrative tasks (p<0.001), earlier hospital discharges (p<0.001), unrealistically high expectations by others (p<0.001), and adverse publicity from the media (p<0.001).  Rural GPs had significantly better scores relating to variety and autonomy (variety of interesting things (p=0.002), decision about how I do my job (p=0.003) and what I do at work (p=0.006).  Both groups reported similar mean positive job attribute statements. Still, they agreed significantly less with each of the four negative job attribute statements (p<0.001) (required to do unimportant tasks, do not have time to carry out all work, must work very intensively, must work fast).  Rural female GPs reported higher mean job satisfaction than male rural GPs (p<0.001).  Intentions to reduce work participation.   - Rural GPs were more likely to anticipate that they would work abroad or leave medical work entirely in the next 5 years. | - Investigate why rural GPs have a higher intention to leave despite higher job satisfaction. - Explore the impact of the new GMS contract on rural GP job satisfaction and retention. | High  This study provides a comprehensive explanation of the methodology, results, and resulting implications. The authors employed validated measures, utilised appropriate statistical analyses, and drew on a nationally representative sample.  The study effectively addresses potential confounders and biases; although causal inferences cannot be made, the identified associations are meaningful and provide a foundation for further research. |
| (Latham et al., 2025)  Scotland  Funded through a grant (HIPS/19/37) from the Scottish Government's Chief Scientist Office and NHS Highland. | To qualitatively investigate GPs’ decisions to move or stay in rural areas by exploring areas that can promote work engagement. | 44 GPs (n=44, female n =26).  **Role Breakdown**:  GP Partners: 20 (2 were single-handed).  Salaried GPs: 11.  Locum GPs: 7.  Trainee GPs: 2.  Retired: 1.  Other (GP in an extended role): 1.  **Experience**:  Current rural practice (n= 42).  Prior rural experience (n = 2). | Qualitative research with semi-structured interviews was conducted in 2020 via video call or telephone.  The Areas of Worklife Scale (AWS) was used to structure data.  Themes included:  Workload.  Control.  Reward.  Community.  Fairness.  Values. | Burnout Factors (Barriers to Staying or Moving Rurally):   1. Workload:   High workload and out-of-hours commitments led to stress and exhaustion.  Emergency care responsibilities without adequate support or training created fear and pressure.  The mismatch between rural and urban GP training resulted in some doctors feeling unprepared for rural work.   1. Clinical Isolation:   Lack of professional networks and colleagues increased feelings of isolation.  In some cases, this led to leaving rural roles.   1. Personal and Family Factors:   Lack of partner employment opportunities.  Challenges with rural infrastructure, including housing and schooling.  Concerns about balancing family life with professional demands.   1. Perceived Unfairness:   Rural GPs felt undervalued by policymakers and management.  Financial incentives were insufficient, and salary disparities led to frustration.  Engagement Factors (Facilitators for Staying or Moving Rurally):   1. Professional Autonomy:   Rural settings allowed GPs more flexibility and control over their work.  The ability to spend more time with patients was highly valued.   1. Supportive Communities:   Professional networks and peer support reduced isolation and increased job satisfaction.  Many GPs appreciated the sense of community and connection in rural areas.   1. Alignment with Professional Values:   Continuity of care was a core value, which rural settings allowed GPs to fulfil.  GPs appreciated their ability to make meaningful contributions to their communities.   1. Rural Lifestyle:   Many GPs valued the rural lifestyle for themselves and their families, emphasising outdoor activities and a tranquil environment. | - Define the unique role and challenges of rural primary care. - Involve communities in physician recruitment and retention efforts. - Provide accessible pathways for maintaining rural clinical skills. - Evaluate existing interventions to build an evidence base for policy. - Ensure health policy decisions are equitable for rural primary care. | Moderate to High.  The study has a clear focus, a rigorous qualitative framework and comprehensive ethical considerations.  However, the absence of reflexivity and the challenges inherent in secondary data analysis slightly weaken the study’s interpretive depth. |
| (Harry et al., 2024)  United States  Funding from Essentia Health. | Explore (1) whether burnout and contributing work conditions differed between rural and urban clinicians and between physicians and advanced practice clinicians (APCs: NPs, PAs, clinical nurse specialists, midwives, certified registered nurse anaesthetists) in an Upper Midwestern health care system; and (2) contributors to burnout in family practice clinicians. | Full Sample  Physicians  Urban (n=419, 46.1% male)  Rural (n=170, 57.1% male)  APCs  Urban (n=317, 16.1% male)  Rural (n=179, 11.7% male)  Family Practice Sample  Physicians  Urban (n=47, 36.2% male)  Rural (n=59, 49.2% male)  APCs  Urban (n=39, <5%% male)  Rural (n=78, 7.7% male) | An exploratory study for the evaluation of burnout.  Mini Z instrument version 1.0, with 2 items on the frequency of encountering negative experiences at work due to gender or race, and an item on intent to leave work in the next 2 years. | Rural and urban clinician burnout rates were comparable (45% vs 47%).  In multivariate models for rural clinicians, factors that increased burnout odds: stress (OR: 8.53, 95% CI: 4.09 to 17.78, P<.001), lack of workload control (OR: 3.06, 95% CI: 1.47-6.36, P=0.003), busy/chaotic environments (OR: 2.53, 95% CI: 1.29- 4.99, P=0.007), intent to leave (OR: 2.18, 95% CI: 1.06-4.45, P=0.033).  Multivariate binary logistic regression analyses showed no significant differences in burnout based on gender, clinician role, or rurality among family practice clinicians.  Part-time work protected against burnout for family practice and rural clinicians, but not urban clinicians. | Work Environment & Conditions:   - Address key burnout drivers (stress, workload control, chaos) to improve rural work environments and aid recruitment. - Prioritise stress reduction, particularly in family practice settings. - Promote part-time work as a protective factor against burnout.   Assessment & Intervention:   - Implement systematic screening and intervention to mitigate the impact of burnout. - Utilise the Mini Z measure as a validated tool for burnout assessment.   Future Research:   - Conduct further research to confirm the findings and identify additional drivers of burnout in rural settings. | High  The study presents a precise aim, an appropriate design that incorporates ethical considerations, and comprehensive data collection and analysis. However, there are some minor areas for improvement, including the need to mention reasons for non-participation and the handling of missing data, which limit generalisability. |
| (Ward et al., 2021)  United States  No funding reported. | Determine whether burnout rates in FPs differed by  rural and urban practice location. | Family physicians (FPs)  Rural (n = 408, 35.8% female, 57.1% were 50 years and older).  Urban (n = 2,332, 33.9% female, 52.3% were 50 years and older). | Cross-sectional study   - Use of the Family Medicine Certification examination registration questionnaire. (Mandatory component of examination) - 2 validated single-item questions to measure the emotional exhaustion and depersonalisation domains of burnout that correlate highly with these subscales of the MBI. - Mini-Z.   Scope of Practice for Primary Care (SP4PC) score | Rural FPs had a non-significantly higher burnout rate than urban FPs (45.1% vs 43.0%).  Burnout was more common among younger individuals and females.  No rural/urban differences were found between job satisfaction, practice environment, workload, and job stress; however, all of these characteristics were associated with burnout.  Rural FPs had a significantly broader scope of practice (15.5 vs 12.5 SP4PC score).  Rural FPs (33.3%) reported being a faculty member.  Rural FPs are primarily employed in hospital-owned practices (40.9% vs 32.9%) followed by group private practice (20.8% vs 23.7%)  FPs 60 years and older were less likely to be burned out than their middle-aged colleagues (15.3% vs 39.7% of  40- to 49-year-olds and 34.7% of 50- to 59-year-olds)  Factors associated with burnout:  lack of control over workload,  less time for documentation,  chaos and stress.  Vulnerable patients | - Recognise that burnout drivers are common across both rural and urban settings. - Focus interventions on organisational factors, such as improving physician autonomy, reducing workplace chaos, aligning values with leadership, and enhancing team support. - Acknowledge that healthcare system acquisitions can worsen burnout by reducing physician independence and creating value conflicts. | Moderate  The study has a clear aim, employs appropriate quantitative methods, and utilises mandatory questionnaires that facilitate statistical analysis.  A limitation of the study is the reliance on self-reported data. |
| (Kuroda et al., 2022)  Japan  No Funding reported | Examine the anxiety of young rural physicians working alone on remote islands. | Young physicians (n=6, average age 30.2 ± 2.67 years, all male) who have completed  their 3-year postgraduate clinical training with general rotation in all specialties. | Qualitative research with semi-structured interviews online.  1. What were your anxieties before going to work on a remote island?  2. What were the new anxieties that you started to feel after going to a remote island?  3 Is there anything else that you thought you should have been more prepared for before going to a remote island?  4 Are there any other measures that we could take to relieve these anxieties? | Solo practice   - Anxiety about handling all fields of medicine alone because of poor access to specialists. - Anxiety about caring for all cases, mild to severe, during 24-h shifts, even on weekends and holidays.   Tight-knit community  Professionalism was compromised when their status obscured the physician-patient relationship as fellow island residents.  Limited human and medical resources  Need to fill leadership roles as the only physician.  Anxiety about performing appropriate triage and making adequate judgment calls with limited access to medical services.  Future career  Anxiety about the incongruence between their status and the treatment they received as sole physicians versus their actual level of experience or competency. | - Address physician anxiety to support professional development. - Increase online peer reflection and direct patient experience. - Use interprofessional collaboration to mitigate resource-related anxiety. - Cultivate physician self-awareness to improve patient care. - Expand participant diversity in future research. | Moderate  The study has a clear aim and an appropriate qualitative method to address the research questions. Data collection and analysis, using the SCAT method, ensure understanding. There is limited discussion on researcher-participant relationships and a small sample size, which may limit the generalisability of the findings. |
| (Islam et al., 2024).  Bangladesh  Funded by UK aid from the UK Government as an output of the SOAS Anti-Corruption Evidence (ACE) research consortium. | Investigate the characteristics and recent experiences of Bangladeshi doctors working in rural facilities and their association with absenteeism. | 308 doctors recruited through convenience sampling who have worked in rural facilities for ten years or more.  Respondents were aged 31–35 (57.8%) and male (53.6%).  47% of the respondents work as medical officers at the assigned hospitals. | Facility-based cross-sectional survey on the doctors’ experiences during their rural postings.  The interviews explored perceptions of what drives absenteeism and views on  potential solutions.  The questionnaire includes:   - sociodemographic characteristics, - educational background, - professional affiliation, - job-related information, - rural placement information. - experience and challenges faced by doctors in their last rural post, - self-assessed financial situation,   self-reported connections to powerful or influential people. | 26% reported not serving their mandatory two-year rural posting uninterruptedly, because of availing training opportunities (65%) and family commitments (41%).  (96%) stated that they faced challenges due to:   - physically unsafe environment (73%), - verbal abuse (70%) - staff absenteeism (50%).   Mitigating factors:   - family (39.8%) - local influential people (38.8%) - social networks (colleagues) (34.5%) - friend (32.6%)   75% reported a doctor shortage in the facilities because doctors were not being posted against sanctioned posts (63%), and doctors leaving their posts to pursue higher education (45%).  48% complained that the workload was too high, sometimes unmanageable.  30% were not involved in private practice during their rural postings.  63% mentioned not staying in the residence provided by the authority most of the time, and over three-quarters (76%) of respondents’ families did not remain in the residence provided because of lack of security (77%), lack of basic amenities (72%), and a lack of convenient transport services (40%). | - Implement policies to protect doctors from community threats. - Support physicians who have weak social or professional networks. - Revise training policies to encourage, not penalise, rural service. - Prioritise supportive management over punitive absenteeism measures. - Strengthen community linkages for physician safety and support. - Offer flexible, penalty-free training opportunities to improve retention. | Moderate  The study has a clear goal and appropriate methods, including ethical considerations to address the research questions, and relevant data collection and analysis that allow for practical recommendations to be made. Some limitations include how researcher bias was managed and aspects of the recruitment strategy, e.g. a convenience sample of urban-based doctors  and data collection methods, which limited generalisability and introduces a high risk of selection, recall, and survivorship bias. |
| (Naher et al., 2022)  Bangladesh  No funding reported | Understand the socio-economic and political factors that contribute to absenteeism among junior doctors in rural health facilities in Bangladesh. | Young doctors with a minimum of two years' experience (n=30), age range 27-58 years.  Medical officer (n=18)  Residential medical officer (n=5)  Senior doctors (hospital director, assistant director, professor, and consultant (n=7) | Cross-sectional qualitative research design and critical realist approach using a semi-structured interview guide, which focused on eliciting a rich description of experiences of working and living in rural positions. | Drivers of absenteeism   - Chaotic and stressful environment due to insufficient doctors for the number of patients, and patients are unwilling to wait. - Political pressure to issue false medical certificates. - Assaults are occurring within health facilities and the local community. - Poor accommodation and a lack of security, particularly for women who choose to live outside and travel into work every day. - Shortages and stock-outs of medicine, equipment, and other basic supplies. - They need to recruit additional staff and pay their salaries out of their own pockets to keep the facility clean and manage the queues of patients. - Long working hours - Lack of time to study to progress in their careers. - Need to travel to a city for both preparatory classes and the exams. - A biometric system to monitor attendance, and if unauthorised absence for more than three consecutive days was expected to result in formal action, except for doctors with political connections. - Difficulty in taking leave, and said that they often had to work extremely hard to cover for absent colleagues and vacancies.   Drivers to work in rural areas.  Good social relationships with locally powerful families | - Address systemic health and political issues that drive physician absenteeism. - Improve rural infrastructure, work environments, and career pathways. - Address practical challenges faced by rural doctors, including inadequate housing and limited educational opportunities. - Implement anti-corruption strategies to incentivise rural retention. - Use findings to guide national policy on absenteeism and healthcare access. - Inform health coverage extension policies in other low- and middle-income countries. | Moderate  The study has a well-defined objective, employs suitable methodologies that incorporate ethical considerations to tackle the research questions, and conducts appropriate data collection and analysis to facilitate the formulation of practical recommendations. The study's limitations encompass the management of bias and the absence of information regarding recruitment and data collection as well as researcher reflexivity. |
| (Hain et al., 2021)  South Africa  Supported in part by the National Research Foundation of South Africa | To determine the prevalence of burnout, depression and anxiety in doctors working in rural district hospitals in northern KwaZulu-Natal (KZN) Province, SA, and to explore the associated sociodemographic and rural work-related factors. | Doctors (n = 96, 47.9% male)  Community service medical officer (CSMOs) (32.5%)  Medical officer <5 years’ experience (18.1%)  Medical officer ≥5 years’ experience (43.4%)  Other (6%). | Quantitative descriptive cross-sectional study.  Four self-report questionnaires:   - a sociodemographic and occupational profile questionnaire - MBI - PHQ-9 - GAD-7   Participants were also asked to choose the six most important factors (from a list of 24) that contributed to stress and the three most important factors (from a list of 14) that would prevent stress build-up. | MBI (n = 89): 68.5% were assessed as having burnout.  PHQ-9 (n=87) 35.6% were positive for depression  GAD-7 (n=86) 23.3% for anxiety.  Associations between burnout, depression and anxiety and sociodemographic and occupational profile variables   - Burnout alone was significantly associated with gender and anxiety, with 84.8% (n=39) of females having high scores in EE or DP. - Burnout and anxiety were both significantly associated with occupational rank. - Burnout, depression and were all significantly associated with doctors planning on leaving the public sector within the next 2 years. - Anxiety was associated with unwillingness to work in a rural setting. - Anxiety and depression were associated with country of qualification, with SA-qualified doctors reporting a higher rate.   Perception of contributory and preventive factors  Factors contributing to stress were:   - difficulty referring patients (n=58), - staff shortages (n=41), - lack of equipment (n=41), - lack of management support (n=39), - balancing work and personal life (n=35), - difficulty with language/culture (n=30). - lack of specialist back-up support (n=30).   Factors preventing stress build-up:  improving recruitment (n=36),  management skills/systems (n=32)  staff relationships (n=27).   - 75% (n=57) reported that an independent service provider unaffiliated with their hospital would be their preferred source of support for burnout, depression, or anxiety. | - Shift policy focus from recruitment to the retention of rural doctors. - Prioritise evidence-based solutions to prevent burnout and improve retention. - Develop multi-level interventions addressing both organisational and individual factors. - Provide targeted support for high-risk groups, such as Community Service Medical Officers (CSMOs) and female doctors. - Conduct further research on burnout, depression, and anxiety in other rural contexts. | Moderate  The study provides detailed information on a public health issue using validated tools and comprehensive reporting. However, limitations related to the sampling method (a convenience sample with a low response rate) introduce potential biases, reduce generalisability, and hinder the ability to establish causality, thereby reducing the overall strength of the evidence. |
| (Purbrick et al., 2024)  South Africa  Partial financial support was received from the University of the Witwatersrand School of Clinical Medicine Graduate Studies and Research Committee MMED Fund. | Determine the prevalence of burnout among community service doctors in South Africa (SA), and the potential contributory and protective factors. | Community service doctors (a junior doctor who has just completed a 2-year  period of internship) (n = 208).  Median age (27 years old (IQR 26–  28)  Work setting: 44% in district hospitals.  Geographical distribution: Majority from Gauteng and Western Cape provinces | Quantitative, descriptive cross-sectional study.  Burnout: MBI-HSS (MP) Depression: PHQ-9 | Burnout (83%)  High EE (89%)  High DP (94%)  Low PA (97%)  **Contributing factors for:**  EE   - mental illness (OR 4.25, 95%CI [1.31,21.6]), - financial difficulty (OR 5.14, 95%CI [1.27,47.0]), - strongly disagree with the volume of patients they are expected to see is manageable (OR 9.87, 95%CI [1.07, 1.309]).   DP   - doing > 30 hours of actual overtime per week (OR 39.9 95%CI [1.80,6.298,41]). - individual calls lasting 24–30 hours (OR 7.69, 95%CI[1.15,13.6]).   PA  Female (OR 4.55, 95%CI [1.07, 21.0]).  **Protective factors for:**  EE   - working at a district hospital (OR 3.86, 95%CI [1.14,13.6]) - manageable volume of patients (OR 0.29, 95%CI [0.09, 0.86])   DP   - satisfied with their decision to study medicine and become a doctor (OR 0.11 95% CI [0.01, 0.55]) - manageable volume of patients expected to see (OR 0.17, 95%CI [0.03, 0.91]) - disagree that they are required to stay at work after the recommended end of work time (OR 0.09, 95%CI [0.00,0.80]).   PA   - having >2 children (OR 0.03, 95%CI [0.00,0.47]), - taking over-the-counter (OTC) medication (OR 0.15, 95%CI [0.03,0.87]), - talking to colleagues as a coping mechanism (OR 0.13, 95%CI [0.09, 4.61]) - feel supported by hospital management (OR 0.00, 95%CI [0.00, 0.14])   **Consequences**  EE   - leaving the government sector (private sector, different field, sabbatical) (OR 3.38, 95%CI [1.37, 9.35]) - diagnosed with a mental illness (OR 4.25, 95%CI [1.31,21.6]).   DP   - leaving the government sector (for the private sector, a different field or sabbatical) (OR 3.57, 95%CI [1.12, 14.5]), - using alcohol as a coping mechanism (OR 11.5, 95%CI [1.48, 1.479])   No significant associations with PA  **Depression** (49%)   - high EE score (98%)   high DP score (97%) | - Improve access to mental health care for community service doctors.  - Implement exercise programs at work.  - Strictly cap working hours.  - Address systemic issues like resource and staff shortages.  - Enhance mental health education to raise awareness about burnout.  - Conduct further research on the relationship between burnout and depression.  - Conduct qualitative research on the causes of burnout.  - Investigate the impact of burnout on patients.  - Use probability sampling in future studies to confirm findings. | Moderate  The study meets most of the criteria for methodological quality but has some limitations due to the convenience sampling strategy and potential nonresponse bias, all of which limit generalisation. |

**Abbreviation**: DASS‐21, Depression and Anxiety Stress Scales, 21‐item version; CBI, Copenhagen Burnout Inventory; COPSOQ‐II, Copenhagen Psychosocial Questionnaire, second version; WAI, Work Ability Index; MBI, Maslach Burnout Inventory; MBI-GS, MBI-General Survey; MBI-HSS, MBI-Human Services Survey; MBI‑HSS (MP), ‑ MBI-HSS for Medical Personnel; ISO, International Organization for Standardization; JMO, Junior medical officer; PHQ-9, Patient Health Questionnaire-9, GAD-7, Generalized Anxiety Disorder 7-item questionnaire; ERI, Effort-Reward Imbalance ERI questionnaire, GHQ-12, Chinese version 12-item General Health Questionnaire, SPS-6, Stanford Presenteeism Scale 6-item; PSS-4, Perceived Stress Scale; WFC, Work-Family Conflict Scale; BFI, Big Five Inventory; TAS-20, Toronto Alexithymia Scale; UWES, Utrecht Work Engagement Scale, SCL-90, Symptom Checklist 90; OLBI, Oldenburg Burnout Inventory
