# Supplementary material for: Stress and retention challenges among rural and regional physicians: a mixed-methods systematic review and framework for action
Source: J Public Health (Oxf). 2026 Feb 15;48(2):572–81. doi: 10.1093/pubmed/fdag011 (PMC13223598; doi:10.1093/pubmed/fdag011)
Supplement: Supplemental_Data_S4_fdag011 [file supplemental_data_s4_fdag011.pdf]

Supplemental Data S4: Critical appraisal of selected papers using the QuADS appraisal tool for multi-method studies.

References	Criterion 1: Theory /Concept	Criterion 2: Aim/s	Criterion 3: Setting / Population	Criterion 4: Study design	Criterion 5: Sampling	Criterion 6: Rationale for choice of data collection tool/s	Criterion 7: Format and content of data collection tool	Criterion 8: Description of data collection procedure	Criterion 9: Recruitment data	Criterion 10: Justification for analytic method	Criterion 11: Analysis appropriate	Criterion 12: Stakeholders considered	Criterion 13: Strengths and limitations
Ji J, Han Y, Li R, Jin H, Yin C, Niu L, et al. The role of effort-reward imbalance and depressive symptoms in the relationship between long working hours and presenteeism among Chinese village doctors: a moderated mediation model. BMC Psychiatry. 2023;23(1):497.	3	3	3	2	2	3	3	3	3	2	3	0	3
Wang A, Tang C, Zhou L, Lv H, Song J, Chen Z, et al. How surface acting affects turnover intention among family doctors in rural China: the mediating role of emotional exhaustion and the moderating role of occupational commitment. Hum Resour Health. 2023;21(1):3.	3	3	3	2	2	3	3	3	3	2	3	1	3
Yan W, Sun G. Income, workload, and any other factors associated with anticipated retention of rural doctors? Prim Health Care Res Dev. 2022;23:e12.	2	3	3	3	1	0	2	3	3	0	3	0	2
Zhang X, Bian L, Bai X, Kong D, Liu L, Chen Q, et al. The influence of job satisfaction, resilience and work engagement on turnover intention among village doctors in China: a cross-sectional study. BMC Health Serv Res. 2020;20(1):283.	3	3	3	3	3	3	3	3	3	2	3	0	3
Zhang X, Bai X, Bian L, Wang M. The influence of personality, alexithymia and work engagement on burnout among village doctors in China: a cross-sectional study. BMC Public Health. 2021;21(1):1507.	3	3	3	3	3	3	3	3	3	2	3	0	3
Zhao X, Liu S, Chen Y, Zhang Q, Wang Y. Influential Factors of Burnout among Village Doctors in China: A Cross-Sectional Study. Int J Environ Res Public Health. 2021;18(4).	3	3	3	3	1	3	3	3	3	1	3	0	3
Zhu X, Chen Y, Liao X. Factors underlying burnout among rural village physicians in Southwestern China. Health Care Sci. 2023;2(4):233-41.	3	3	3	3	1	3	3	3	3	2	3	0	3
Hadley A. Migration and professional mobility: Rural attraction and retention of South African educated physicians. Soc Sci Med. 2024;350:116884.	3	3	3	3	3	3	3	3	3	3	3	0	3

Hansen N, Jensen K, MacNiven I, Pollock N, D'Hont T, Chatwood S. Exploring the impact of rural health system factors on physician burnout: a mixed-methods study in Northern Canada. BMC Health Serv Res. 2021;21(1):869.	3	3	3	3	1	3	3	3	3	3	3	0	3
Jolicoeur J, DeMiglio L, LN RK, Orrantia E. Why they leave: Small town rural realities of northern physician turnover. Can J Rural Med. 2022;27(1):22-8.	3	3	3	3	3	3	3	2	3	2	3	0	3
Lesperance S, Anaraki NR, Ashgari S, Churchill A. Systemic challenges and resiliency in rural family practice. Can J Rural Med. 2022;27(3):91-8.	3	3	3	3	3	3	3	3	3	3	3	0	3
Clough BA, Ireland MJ, Leane S, March S. Stressors and protective factors among regional and metropolitan Australian medical doctors: A mixed methods investigation. J Clin Psychol. 2020;76(7):1362-89.	3	3	3	3	1	3	3	3	3	3	3	0	3
Fitzpatrick M, Garsia K, Eyre K, Blackhall CA, Pit S. Emotional exhaustion among regional doctors in training and the application of international guidelines on sustainable employability management for organisations. Aust Health Rev. 2020;44(4):609-17.	3	3	3	3	1	3	3	3	3	2	3	0	3
Eaton-Hart JH, Gillies JC, Mercer SW. How do the working lives of general practitioners in rural areas compare with elsewhere in Scotland? Cross-sectional analysis of the Scottish School of Primary Care National GP Survey. RRH. 2022;22(3):7270.	3	3	3	3	3	3	3	3	3	3	3	0	3
Latham HA, Maclaren AS, De Kock JH, Locock L, Murchie P, Skea Z. Exploring rural Scottish GPs' migration decisions: a secondary qualitative analysis considering burnout. Br J Gen Pract. 2025;75(752):e187-e94.	3	3	3	3	3	3	3	3	3	3	3	0	2
Harry ML, Sudak NL, Engels MJ, Horn KK, Dean K, Poplau S, et al. Physician and Advanced Practice Clinician Burnout in Rural and Urban Settings. J Am Board Fam Med. 2024;37(1):43-58.	3	3	3	3	2	3	3	3	3	3	3	0	3
Ward ZD, Morgan ZJ, Peterson LE. Family Physician Burnout Does Not Differ With Rurality. J Rural Health. 2021;37(4):755-61.	3	3	3	3	3	3	3	3	3	2	3	0	3
Kuroda K, Kuroda M, Ohta R. Sources of anxiety in young rural physicians working alone on remote islands: A qualitative study. J Gen Fam Med. 2022;23(2):128-32.	2	3	3	3	1	3	3	3	3	3	3	0	3

Islam MR, Angell B, Naher N, Islam BZ, Khan MH, McKee M, et al. Who is absent and why? Factors affecting doctor absenteeism in Bangladesh. PLOS Glob Public Health. 2024;4(4):e0003040.	3	3	3	3	1	3	3	2	2	3	3	2	3
Naher N, Balabanova D, McKee M, Khan MH, Roy P, Ahmed SM, et al. Absenteeism among doctors in the Bangladesh health system: What are the structural drivers? SSM - Qual Res Health. 2022;2.	3	3	3	3	2	3	3	2	2	3	3	2	2
Hain S, Tomita A, Milligan P, Chiliza B. Retain rural doctors: Burnout, depression and anxiety in medical doctors working in rural KwaZulu-Natal Province, South Africa. S Afr Med J. 2021;111(12):1197-204.	3	3	3	3	1	3	3	3	3	2	3	0	3
Purbrick GM, Morar T, Kooverjee J. Burnout among community service doctors in South Africa. Afr J Prim Health Care Fam Med. 2024;16(1):e1-e9.	3	3	3	3	1	3	3	3	3	2	3	0	3
