# Supplementary material for: Stress and retention challenges among rural and regional physicians: a mixed-methods systematic review and framework for action
Source: J Public Health (Oxf). 2026 Feb 15;48(2):572–81. doi: 10.1093/pubmed/fdag011 (PMC13223598; doi:10.1093/pubmed/fdag011)
Supplement: Supplemental_Data_S5_fdag011 [file supplemental_data_s5_fdag011.docx]

**Supplemental Data S5:** Reasons for exclusion on full-text screening**.**

1. Alzaher HH, Tharwatabdullahalmanasef, Alqudaihi OA, et al. Diagnosing the causes of work stress among doctors and nurses working in Hospitals in the Eastern Province of Saudi Arabia: An Analytical Comparative Study. Article. *J Cardiovasc Dis Res*. 2022;13(4):331-349. doi:[10.31838/jcdr.2022.13.04.41](file:///C:\Users\jmoensco\Dropbox%20(UNE%20Enterprise)\Joelle\GP%20in%20rural%20area\10.31838\jcdr.2022.13.04.41)

*The Eastern Province of Saudi Arabia, being the third most populous province (with more than 4.9 million inhabitants), was not included in our analysis.*

1. Chen J, Wang Y, Du W, Liu S, Xiao Z, Wu Y. Analysis on the relationship between effort-reward imbalance and job satisfaction among family doctors in China: a cross-sectional study. *BMC Health Serv Res*. Aug 4 2022;22(1):992. doi:[10.1186/s12913-022-08377-5](file:///C:\Users\jmoensco\Dropbox%20(UNE%20Enterprise)\Joelle\GP%20in%20rural%20area\10.1186\s12913-022-08377-5)

*The study’s context is family doctor contract services in a highly urbanised setting, which does not align with the review's focus on small-populated areas.*

1. Gebska Kuczerowska A, Rozenek H, Frajnt-Dabrowska M, Rabczenko D, Banasiewicz J, Gajda R. Features of personality and professional burnout syndrome of physicians - analysis based on questionnaires studies. *Przegl Epidemiol*. 2020;74(3):531-542. doi:[10.32394/pe.74.46](file:///C:\Users\jmoensco\Dropbox%20(UNE%20Enterprise)\Joelle\GP%20in%20rural%20area\10.32394\pe.74.46)

*The study focuses on medical practitioners from the Mazovian District in Poland without specifying whether these physicians practice in remote, rural, or regional areas.*

1. Humphries N, Creese J, McDermott AM, Colleran G, McDermott C, Byrne JP. 'That's just how medicine is': A remote ethnographic study of Ireland's failure to meet the core work needs of its hospital doctors. *Ssm-Qual Res Health*. Jun 2024;5doi:[ARTN 10039210.1016/j.ssmqr.2024.100392](file:///C:\Users\jmoensco\Dropbox%20(UNE%20Enterprise)\Joelle\GP%20in%20rural%20area\ARTN%2010039210.1016\j.ssmqr.2024.100392)

*The study focuses on hospital doctors in Ireland without specifying remote, rural, or regional areas. Its primary context is the strain on the Irish health system due to the pandemic.*

1. Marcotte LM, Maynard C, Reddy A, et al. Factors associated with primary care physician turnover in the VA. *The American journal of managed care*. 2024;30(7):89527-89527.

*Not available online at the time of extraction.*

1. Naehrig D, Glozier N, Klinner C, et al. Determinants of well-being and their interconnections in Australian general practitioners: a qualitative study. *BMJ Open*. Jul 18 2022;12(7):e058616. doi:[10.1136/bmjopen-2021-058616](file:///C:\Users\jmoensco\Dropbox%20(UNE%20Enterprise)\Joelle\GP%20in%20rural%20area\10.1136\bmjopen-2021-058616)

*While the study includes some general practitioners (GPs) from regional and rural areas in Australia, it does not clearly distinguish between those working in rural or regional areas. Furthermore, the primary study focuses on the determinants of well-being among GPs rather than specific stress-related outcomes such as burnout, stress, anxiety, insomnia, depression, absenteeism, or intention to leave.*

1. Yellowlees P, Coate L, Misquitta R, Wetzel AE, Parish MB. The association between adverse childhood experiences and burnout in a regional sample of physicians. *Academic Psychiatry*. Apr 2021, 2023-05-25 2021;45(2):159-163. doi:<https://doi.org/10.1007/s40596-020-01381-z>

*While the study focuses on a regional sample of physicians in Northern California, it doesn't describe personal and work-related factors associated with stress-related outcomes, specifically in the context of practising in remote, rural, or regional areas.*

1. Zhao D, Gao F, Liu W, Cui JZ. Worrying results on resignation intention for ICU physicians in China: a big data report from 34 provinces. Article. Eur Rev Med Pharmacol Sci. May 2023;27(9):3799-3808. doi: [10.26355/eurrev_202305_32285](file:///C:\Users\jmoensco\Dropbox%20(UNE%20Enterprise)\Joelle\GP%20in%20rural%20area\10.26355\eurrev_202305_32285)

*This study investigates factors influencing resignation intention among ICU physicians practising in major tertiary hospitals across 34 provinces without describing the rural context.*

1. Terry, D.L. and M.J. Woo, Burnout, job satisfaction, and work-family conflict among rural medical providers. Psychol Health Med, 2021. 26(2): p. 196-203.

*While providing the medical providers' survey breakdown, the authors do not make a clear distinction in their analysis.*

1. Al-Haddad, A., et al. (2020). Prevalence of burnout syndrome and its related risk factors among physicians working in primary health care centers of the Ministry of Health, Al Ahsa region, Saudi Arabia, 2018-2019. *J Family Med Prim Care*, *9*(2), 571-579. <https://doi.org/10.4103/jfmpc.jfmpc_743_19>.

*The paper does not provide a distinct model or a separate list of contributing factors for the rural group versus the urban group.*

1. Johnsson, L., & Nordgren, L. (2022). The voice of the self: a typology of general practitioners' emotional responses to situational and contextual stressors [Article]. Scand J Prim Health Care, 40(2), 289-304. <https://doi.org/10.1080/02813432.2022.2097616>.

*The data in the paper are from distinct settings that were aggregated to form a single conceptual model. The paper does not present any findings, themes, or analyses specific to the rural cohort.*
